# High‐throughput serum proteomics for the identification of protein biomarkers of mortality in older men

**DOI:** 10.1111/acel.12717

**Published:** 2018-02-05

**Authors:** Eric S. Orwoll, Jack Wiedrick, Jon Jacobs, Erin S. Baker, Paul Piehowski, Vladislav Petyuk, Yuqian Gao, Tujin Shi, Richard D. Smith, Douglas C. Bauer, Steven R Cummings, Carrie M. Nielson, Jodi Lapidus, for the Osteoporotic Fractures in Men Study (MrOS) Research Group

**Affiliations:** ^1^ Oregon Health & Science University Portland OR USA; ^2^ Biological Science Division Pacific Northwest National Laboratory Richland WA USA; ^3^ Department of Medicine University of California San Francisco CA USA; ^4^ California Pacific Medical Center Research Institute San Francisco CA USA

**Keywords:** aging, biomarker, inflammation, men, mortality, proteomics

## Abstract

The biological perturbations associated with incident mortality are not well elucidated, and there are limited biomarkers for the prediction of mortality. We used a novel high‐throughput proteomics approach to identify serum peptides and proteins associated with 5‐year mortality in community‐dwelling men age ≥65 years who participated in a longitudinal observational study of musculoskeletal aging (Osteoporotic Fractures in Men: MrOS). In a discovery phase, serum specimens collected at baseline in 2473 men were analyzed using liquid chromatography–ion mobility–mass spectrometry, and incident mortality in the subsequent 5 years was ascertained by tri‐annual questionnaire. Rigorous statistical methods were utilized to identify 56 peptides (31 proteins) that were associated with 5‐year mortality. In an independent replication phase, selected reaction monitoring was used to examine 21 of those peptides in baseline serum from 750 additional men; 81% of those peptides remained significantly associated with mortality. Mortality‐associated proteins included a variety involved in inflammation or complement activation; several have been previously linked to mortality (e.g., C‐reactive protein, alpha 1‐antichymotrypsin) and others are not previously known to be associated with mortality. Other novel proteins of interest included pregnancy‐associated plasma protein, VE‐cadherin, leucine‐rich α‐2 glycoprotein 1, vinculin, vitronectin, mast/stem cell growth factor receptor, and Saa4. A panel of peptides improved the predictive value of a commonly used clinical predictor of mortality. Overall, these results suggest that complex inflammatory pathways, and proteins in other pathways, are linked to 5‐year mortality risk. This work may serve to identify novel biomarkers for near‐term mortality.

## 1 INTRODUCTION

Biomarkers may have a variety of biomedical applications, including the identification of individuals at risk of clinical outcomes, improving the evaluation of interventions aimed at modifying outcomes, and the elucidation of biological pathways that contribute to health or disease. In the field of aging, there have been numerous studies aimed at the identification of prognostic biomarkers of mortality (Arbeev, Ukraintseva & Yashin, 2016; Barron, Lara, White & Mathers, 2015). Most have utilized assays to evaluate specific candidate biomarkers believed to reflect relevant biological events, including many related to inflammation (Enomoto et al., 2014; Kabagambe et al., 2011; Reuben et al., 2002; Schnabel et al., 2013; Zuo et al., 2016). The identification of good biomarkers could be useful to identify biological processes associated with mortality and to assess the effectiveness of interventions aimed at reducing mortality or extending health span.

Proteomics is a powerful technology that has been used for biomarker discovery (Huang, Ma, Huang, Li & Nice, 2017). Until recently, the discovery of new biomarkers using proteomic approaches has been limited by methods that are demanding of time and resources and have an inherently low throughput. Thus, studies have been generally restricted to relatively small sample sizes that are inadequate to assess variability on a population scale. New approaches, such as aptamer‐based or antibody‐based affinity proteomics, allow multiplexing and larger sample sizes but are limited to the evaluation of a relatively limited number of candidate proteins (Delfani et al., 2016). The plasma proteome is particularly complex but offers an important window on individual variation and the relationship of proteins to important phenotypes. We have recently developed high‐throughput and sensitive methods that allow a broad assessment of the serum proteome (Baker et al., 2010). Similar pipelines for large‐scale discovery proteomics have been employed in several pioneering studies (Geyer et al., 2016; Surinova et al., 2015). Here, we describe the use of these high‐throughput proteomic methods in a large cohort of older men, including an initial discovery phase followed by a validation phase in an independent subcohort, for the identification of peptides and proteins associated with mortality. We detect proteins previously well documented to be associated with mortality, but also a variety of novel proteins. These results provide unique insight into the biological basis of mortality and illustrate the potential utility of this approach for biomarker discovery.

## 2 RESULTS

The characteristics of the discovery and replication cohorts are shown in Table 1. In the discovery cohort (*N* = 2473), the mean age was 73.6 ± 5.8 years (range 65–99). Among these men, death occurred within 2 years of enrollment in 66 (2.7%), in 119 within 3 years (4.8%), and in 267 within 5 years (10.8%). Few men withdrew from the study: two men within 2 years, 12 within 3 years, and 32 within 5 years. Overall, the cause of death was related to cancer in about one‐thirds at any time point and to other causes in the remaining two‐thirds. The characteristics of the men in the replication cohort were similar (Table 1).

**Table 1:** Cohort characteristics

	MrOS Cohort	Discovery subcohort (random sample)	Replication subcohort (random sample)	Replication subcohort (added deaths^a^)
Cohort Size	5994	2473	533	216
Age (years) mean ± *SD*	73.7 ± 5.9	73.6 ± 5.8	74.2 ± 6.0	78.4 ± 7.0
BMI (kg/m^2^) mean ± *SD*	27.4 ± 3.8	27.4 ± 3.8	27.5 ± 3.8	26.9 ± 4.2
Self‐Reported Health Fair/poor/very poor (%)	14.3	13.6	13.9	30.2
Mortality 2 years (%) proportion ± SE	2.8 ± 0.2	2.7 ± 0.3	1.9^b^ ± 0.6	30.1 ± 3.1
Mortality 3 years (%) proportion ± SE	4.9 ± 0.3	4.8 ± 0.4	4.9 ± 0.9	50.0 ± 3.4
Mortality 5 years (%) proportion ± SE	10.4 ± 0.4	10.8 ± 0.6	10.1 ± 1.3	100^a^

a After initial random sampling, the replication subcohort was enriched with all remaining cases of death (within 5 years) in an independent subcohort of MrOS.

b *p* = .29 for H_0_: mortality_IMS,2 yrs_ ≡ mortality_SRM,2 yrs_ and *p* = .60 for H_0_: |mortality_IMS,2 yrs_ – mortality_SRM,2 yrs_| > 0.6% imply the test of equivalence is indeterminate.

### 2.1 Peptides associated with mortality at 5 years

#### 2.1.1 Discovery phase

**Figure 1:**
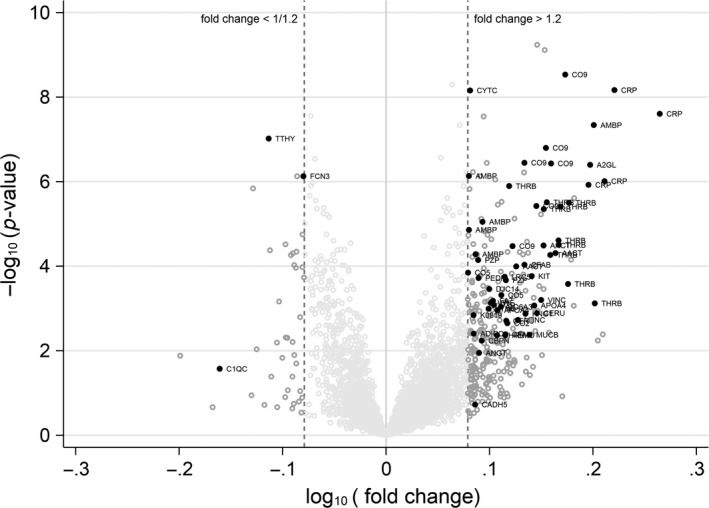
Volcano plot illustrating the associations (fold change) of peptides with mortality. Light gray points represent peptides with initial effect sizes less than 1.2, medium gray points are initial effect sizes ≥1.2 that attenuated below 1.2 under cross‐validation, and the 56 black points (labeled by the matching protein) are effect sizes that were ≥1.2 initially and remained so under cross‐validation (hence *robust*). Also see Section 4.2

The effect sizes of the associations with 5‐year all‐cause mortality are shown in Figure 1. Peptides with robust effect sizes ≥1.2 are represented by black symbols (see Section 4.2). The 56 peptides, representing 31 proteins, that had robust fold changes for 5‐year all‐cause mortality, are listed in Table 2. Peptides were reported in this table only if their effect sizes remained robustly above 1.2 using the cross‐validation approach described in Statistical analyses. Although we report only those peptides with robust associations with mortality, in most cases the 31 proteins were represented by multiple peptides with less robust associations in the same direction. In Supplemental Material, this is represented by the example of the protein AACT. We explored impaired renal function as a possible driver of the associations, but found that adjustments for estimated glomerular filtration rate had negligible impact on the results. As noted in Statistical analyses, robust effects for noncancer mortality at 5 years had complete overlap with all‐cause mortality. Cancer results were somewhat limited by small numbers of deaths; none had robust effect sizes ≥ 1.2.

**Table 2:** Peptides with robust absolute fold change >1.2 for 5‐year all‐cause mortality

Protein	Peptide	Robust fold change (80% CI)
Leucine‐rich alpha‐2‐glycoprotein (A2GL)	CAGPEAVKGQTLLAVAK	1.59 (1.41, 1.80)
Alpha‐2‐macroglobulin (A2MG)	YGRNQGNTWLTAFVLK	1.28 (1.17, 1.41)
	aTVLNYLPK	1.27 (1.16, 1.39)
	PFFVELTMPYSVIR	1.24 (1.16, 1.31)
Alpha‐1‐antichymotrypsin (AACT)	aEQLSLLDRFTEDAKR	1.48 (1.29, 1.69)
	LSLLDRFTEDAKR	1.40 (1.25, 1.58)
	SLLDRFTEDAKR	1.27 (1.17, 1.39)
Leucine‐rich alpha‐2‐glycoprotein (A2GL)	CAGPEAVKGQTLLAVAK	1.59 (1.41, 1.80)
Alpha‐1‐antichymotrypsin (AACT)	aEQLSLLDRFTEDAKR	1.48 (1.29, 1.69)
	LSLLDRFTEDAKR	1.40 (1.25, 1.58)
	SLLDRFTEDAKR	1.27 (1.17, 1.39)
Adiponectin (ADIPO)	YNQQNHYDGSTGK	1.36 (1.24, 1.49)
Protein AMBP (AMBP)	VVAQGVGIPEDSIFTMADRGECVPGEQEPEPILIPR	1.45 (1.29, 1.62)
Angiotensinogen (ANGT)	IDRFMQAVTGWK	1.25 (1.14, 1.37)
Apolipoprotein A‐IV (APOA4)	aDLRDKVNSFFSTFK	1.39 (1.21, 1.60)
	PYADEFKVK	1.27 (1.15, 1.41)
Complement C1q subcomponent subunit C (C1QC)	aFQSVFTVTR	0.60 (0.47, 0.75)
Cadherin‐5 (CADH5)	aYLLKGEYVGK	1.35 (1.14, 1.59)
Carboxypeptidase *N* catalytic chain (CBPN)	aSIPQVSPVR	1.28 (1.17, 1.39)
Ceruloplasmin (CERU)	DIASGLIGPLIICKK	1.39 (1.21, 1.59)
	IYHSHIDAPKDIASGLIGPLIICK	1.27 (1.14, 1.42)
Complement factor B (CFAB)	ALRLPPTTTCQQQKEELLPAQDIK	1.29 (1.17, 1.42)
Complement factor H (CFAH)	aHGGLYHENMR	1.37 (1.22, 1.55)
Complement C2 (CO2)	SQWGKEFLIEK	1.40 (1.24, 1.57)
Complement C5 (CO5)	aRKEFPYRIPLDLVPK	1.41 (1.28, 1.56)
Collagen alpha‐3(VI) chain (CO6A3)	SVEDAQDVSLALTQR	1.33 (1.19, 1.47)
Complement component C9 (CO9)	FTPTETNKAEQCCEETASSISLHGK	1.46 (1.32, 1.62)
	CLCACPFKFEGIACEISK	1.36 (1.22, 1.52)
	aTEHYEEQIEAFK	1.34 (1.21, 1.49)
	aDRDGNTLTYYR	1.32 (1.19, 1.47)
	aKYAFELK	1.26 (1.16, 1.37)
	LSPIYNLVPVK	1.22 (1.12, 1.32)
C‐reactive protein (CRP)	YEVQGEVFTKPQLWP	1.73 (1.45, 2.06)
	aRQDNEILIFWSK	1.58 (1.36, 1.84)
	aGYSIFSYATK	1.53 (1.33, 1.75)
	aESDTSYVSLK	1.48 (1.29, 1.69)
DnaJ homolog subfamily C member 14 (DJC14)	RKEYEMK	1.30 (1.20, 1.41)
Coagulation factor VII (FA7)	LMTQDCLQQSR	1.40 (1.26, 1.56)
Hemopexin (HEMO)	aSHKWDRELISER	1.26 (1.11, 1.43)
Uncharacterized protein KIAA0819 (KO819)	KPLSIPK	1.22 (1.14, 1.32)
Mast/stem cell growth factor receptor (KIT)	HGLSNSIYVFVRDPAK	1.28 (1.15, 1.43)
Kininogen‐1 (KNG1)	aTVGSDTFYSFKYEIK	1.38 (1.22, 1.56)
Leucine‐rich repeat‐containing protein 57 (LRC57)	aKTGVFQLK	1.34 (1.22, 1.48)
Ig mu heavy chain disease protein (MUCB)	SKLICQATGFSPR	1.22 (1.07, 1.40)
Prothrombin (THRB)	KSPQELLCGASLISDR	1.46 (1.23, 1.75)
	WYQMGIVSWGEGCDRDGK	1.43 (1.28, 1.60)
	aENLDRDIALMK	1.41 (1.21, 1.63)
	LKKPVAFSDYIHPVCLPDRETAASLLQAGYK	1.36 (1.21, 1.54)
	aIVEGSDAEIGMSPWQVMLFR	1.34 (1.19, 1.50)
	GQPSVLQVVNLPIVERPVCK	1.30 (1.19, 1.43)
	ITDNMFCAGYKPDEGKR	1.30 (1.18, 1.43)
	PSVLQVVNLPIVERPVCK	1.26 (1.14, 1.40)
	GDACEGDSGGPFVMK	1.25 (1.12, 1.39)
	SPQELLCGASLISDR	1.23 (1.14, 1.32)
	KPVAFSDYIHPVCLPDR	1.22 (1.10, 1.36)
Transthyretin (TTHY)	aTSESGELHGLTTEEEFVEGIYK	0.77 (0.71, 0.84)
Vinculin (VINC)	AVAGNISDPGLQK	1.21 (1.07, 1.36)
Vitronectin (VTNC)	aIYISGMAPRPSLAK	1.29 (1.14, 1.46)
	IYISGMAPRP	1.25 (1.14, 1.38)
von Willebrand factor (VWF)	YLSDHSFLVSQGDREQAPNLVYMVTGNPASDEIK	1.26 (1.16, 1.36)

Note that the LRC57, TTHY, and VTNC effect sizes attenuated to zero in the SRM replication, and for C1QC, the effect size reversed direction of association (negative in IMS, positive in SRM).

a = followed for validation of effect using SRM (21 peptides representing 16 proteins).

#### 2.1.2 Enrichment analyses

**Figure 2:**
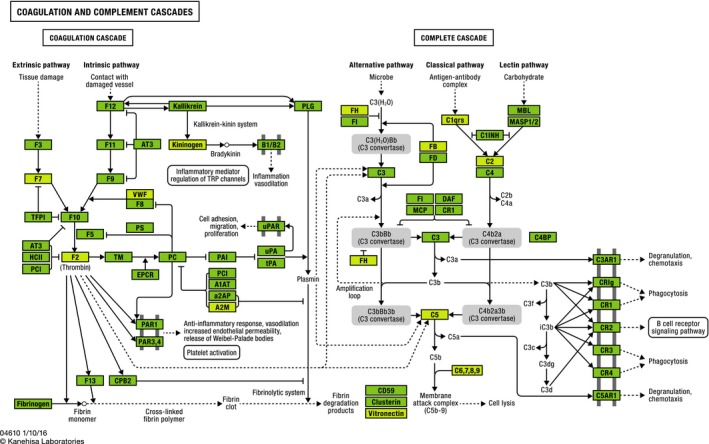
KEGG analysis of mortality‐associated proteins revealed an 11‐fold enrichment in proteins that are members of the “Complement and coagulation cascades” pathway (KEGG term hsa04610). Mortality‐associated proteins are highlighted in yellow. (KEGG Mapper v2.8 released October 20, 2016). Some KEGG names differ from the UniProt names presented in Table 2: A2M = A2MG, C1qrs = C1QC, C2 = CO2, C5 = CO5, C9 = CO9, F2 = THRB, F7 = FA7, FB = CFAB, FH = CFAH, Kininogen = KNG1, Vitronectin = VTNC

Thirty of the 31 proteins represented by the 56 peptides associated with 5‐year all‐cause mortality in the discovery phase were enriched for the KEGG pathway “coagulation and complement cascades” (one of the proteins, K0819, could not be annotated), driven by 11 proteins that included several complement factors, VWF, kininogen, and prothrombin (FDR for enrichment = 2 × 10^−6^, Figure 2). Several of these proteins were also responsible for significant enrichment of the GO biological process terms “regulation of complement activation” and “negative regulation of endopeptidase activity” (Table S1). Half of all mortality‐associated proteins were linked to the GO term “blood microparticle,” whereas approximately 80% of all mortality‐associated proteins were linked to GO terms for extracellular region or exosome (Table S2). Seven of the 30 proteins evaluated had links to the GO molecular function term “receptor binding” (*p* = 1.0 × 10^−4^ but FDR = 12%, Table S3). The 30 mortality proteins had evidence of multiple protein–protein interactions (Figure S1 and http://bit.ly/2lJkCiY).

#### 2.1.3 Replication with SRM

Ninety‐five percent of the peptides assayed by SRM were detected in at least 90% of the replication samples. 82% of peptides had concordant results between discovery and replication for 5‐year all‐cause mortality, which was statistically significant by permutation test (*p* = .05). Seventeen of the 21 peptides selected for SRM replication (from the set of 56 peptides associated with 5‐year all‐cause mortality in the discovery phase [Table 2]) were again associated with 5‐year all‐cause mortality in the same direction and with non‐null effect size. Three more proteins (LRC57, TTHY, and VTNC) had effect sizes that were substantially attenuated compared to those in the discovery phase, and fell within the SRM null window. In one case (C1QC), the effect size was non‐null in SRM but the direction of association was reversed (negative in IMS, positive in SRM).

Peptide associations with cancer mortality failed to replicate in the SRM cohort, but the peptides that showed robust associations with both all‐cause and noncancer mortality in the IMS cohort tended to maintain those associations in the SRM cohort at the same rate as for all‐cause mortality (i.e., approximately 80% agreement).

### 2.2 Peptides associated with mortality at 2 and 3 years

The numbers of deaths were limited after 2 and 3 years of follow‐up, and thus, these analyses are exploratory. The 46 peptides significantly associated with mortality at 2 and 3 years in the discovery phase that were also concordantly associated with mortality in the replication phase are shown in Table 3.

**Table 3:** Peptides associated with mortality in discovery and replication phases for 2‐year or 3‐year all‐cause mortality phenotypes. The 5‐year mortality results for these peptides are included for comparison purposes

Protein	Peptide	Robust fold change (95% CI)		
		2‐year mortality	3‐year mortality	5‐year mortality
A2MG	TVLNYLPK	1.26 (0.93, 1.71)		1.27 (1.11, 1.45)
AACT	EQLSLLDRFTEDAKR	1.36 (1.03, 1.81)		1.48 (1.21, 1.82)
CAH1	ADGLAVIGVLMK	1.21 (0.71, 2.07)		
CFAB	GDSGGPLIVHKR	1.24 (0.89, 1.71)		
CNDP1	HLEDVFSK		0.75 (0.61, 0.91)	
CO4A	DSSTWLTAFVLK	1.36 (1.11, 1.66)	1.23 (1.04, 1.47)	
CO5	RKEFPYRIPLDLVPK		1.38 (1.10, 1.72)	1.41 (1.21, 1.64)
CO9	DRDGNTLTYYR	1.66 (1.14, 2.41)	1.22 (0.99, 1.50)	1.32 (1.12, 1.55)
	KYAFELK	1.33 (0.98, 1.80)		1.26 (1.11, 1.43)
CRP	ESDTSYVSLK	1.40 (1.01, 1.95)		1.48 (1.19, 1.82)
	GYSIFSYATK	1.37 (1.01, 1.88)		1.53 (1.24, 1.88)
GELS	DPDQTDGLGLSYLSSHIANVER		0.81 (0.63, 1.05)	
HYI	IHLMAGR	1.47 (0.70, 3.12)	1.56 (0.87, 2.81)	
ITIH2	KFYNQVSTPLLR		0.76 (0.57, 1.03)	
	MKQTVEAMK		1.50 (1.08, 2.07)	
K2C1	SLDLDSIIAEVK^a^	1.66 (1.22, 2.27)		
KV206	FSGSGSGTDFTLK		0.81 (0.64, 1.03)	
PLMN	EPLDDYVNTQGASLFSVTKK		1.21 (0.98, 1.51)	
SAA4	SGKDPDRFRPDGLPK	1.22 (0.90, 1.64)	1.39 (1.07, 1.81)	
SODE	AVVVHAGEDDLGR	0.70 (0.48, 1.02)		
THRB	ETAASLLQAGYK	1.59 (1.04, 2.42)		
TTHY	SYSTTAVVTNPKE	0.73 (0.48, 1.10)		
	TSESGELHGLTTEEEFVEGIYK^b^	0.81 (0.60, 1.09)	0.75 (0.59, 0.93)	

a Concordant at 2 years but discordant at 3 years.

b Concordant at 2 years and 3 years but attenuated at 5 years.

However, of the 46 peptides selected for replication testing from the 2‐ and 3‐year mortality associations, the 23 that retained associations in the replication phase represent a proportion that would not be atypical even if the SRM associations were independent of the discovery associations (*p* = .48 for 2‐year peptides and *p* = .27 for the 3‐year peptides). Nevertheless, eight of these peptides (six proteins) were also found to be significantly associated with 5‐year mortality, potentially reinforcing their validity as biomarkers at earlier time points. Seven of these eight peptides are related to inflammatory pathways.

### 2.3 Use of a peptide signature to predict mortality

**Figure 3:**
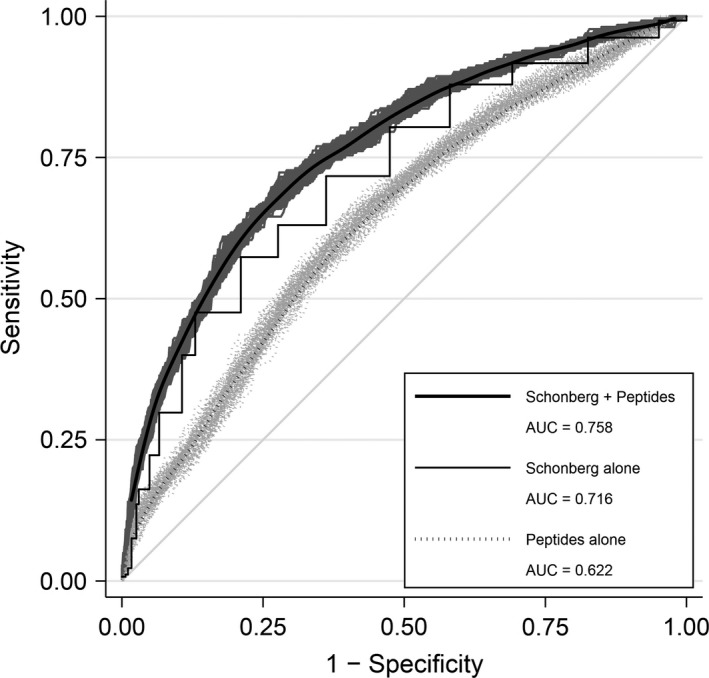
Receiver operating characteristics (ROC) analyses predicting all‐cause mortality: models include peptide signature, Schonberg index, and peptide signature + Schonberg index. The light gray bands show the ROC curves for each of the best‐fitting signatures; the dark gray lines inside the bands represent the average ROC curve for each model. The jagged dark gray line without bands is the ROC curve for the Schonberg index (a step function because the index can take on only a small discrete range of values)

The average ROC curves from the 5‐peptide subset models (see Section 4.2) are shown in Figure 3. The relative overall contribution of each peptide to the best‐fitting models is shown in Table S4. The peptide signature predicted mortality (AUC 0.622). We compared those results to the value of a clinical index for predicting near‐term mortality among community‐dwelling adults aged 65 and older, the Schonberg Mortality Index, that consists of age, BMI, smoking status, and several clinical markers of poor health (Schonberg, Davis, McCarthy & Marcantonio, 2009, 2011) (AUC 0.716). The combination provided the best prediction (AUC 0.758, *p* vs. Schonberg alone = 0.008), suggesting that the peptide signature and the Schonberg index incorporate independent information about the likelihood of death at 5 years.

## 3 DISCUSSION

High‐throughput proteomic analysis of population‐based study samples provides the opportunity to identify biomarkers for important health outcomes and to explore biological pathways that might be involved in health and disease. Using those novel methods, we identified serum peptides that are associated with mortality over 5 years in older, community‐dwelling men. These peptides pointed to a number of proteins with interesting biological implications. In addition to proteins well known to be related to the risk of mortality and cardiovascular disease (e.g., CRP, adiponectin, angiotensinogen, and prothrombin), a variety are involved in inflammation, as well as others related via as of yet unknown mechanisms. Most of the associations between peptide abundance and mortality appeared to arise from noncancer deaths. It is interesting that nearly all the associations were increases in abundance for those who died. The one‐sidedness of the effects may suggest a broad type of biological change, such as cell death or senescence in addition to inflammation.

The search for biomarkers of important health outcomes has been a biomedical research priority. Biomarkers can provide tools for prediction and diagnosis, insight into pathophysiology, and targets for the development of therapeutics. To our knowledge, this study represents the largest proteomic effort to discover biomarkers of biomedical outcomes. Previous proteomic analyses have been limited to smaller numbers of participants and to the assessment of specific candidate proteins or other compounds. Our approach offers an unbiased opportunity to explore large numbers of peptides/proteins in order to identify those associated with outcomes. In addition, we utilized targeted proteomics to replicate peptides identified in the discovery phase, and thus to provide more confidence in their association with mortality.

One major goal of these analyses was to identify potentially useful biomarkers of 5‐year mortality. As proteomic measurements are of peptides, we intentionally report the candidate *peptide* biomarkers. But, in addition, we were interested in exploring the biological bases for these associations, and thereby the biology of mortality. Therefore, our approach also includes a protein‐centric component that incorporates methods providing high confidence in the roll up from peptides to proteins. As above, the essential analytical method (mass spectrometry) is peptide based, and more importantly, the validation step we utilized (SRM) is designed to detect specific peptides. Thus, we believe that reporting these peptides most appropriately reflects the experimental approach. Moreover, we envision that SRM (or a similar method) will become routine in the measurement of multiple peptide biomarkers that predict outcomes (e.g., mortality), and so, reporting the peptides employed in SRM is necessary. Of course, the peptide biomarkers can be interpreted in light of their precursor proteins and we have been diligent in ensuring the peptide‐to‐protein identification step is robust. Our analyses included only peptides that could be unambiguously linked to a protein via established human protein sequence databases. To best ensure the linkages, we employed a conservative confidence scoring system in identifying proteins from peptides. Our methods have been extensively described (Nielson et al., 2017). The number of identified peptides that could not be unambiguously rolled up to proteins (and were thus excluded from consideration for SRM validation) was very small (~2%).

The fact that many peptides were associated with the risk of death as much as 5 years in the future is not only biologically interesting but also supports the potential usefulness of population proteomic approaches to identify peptides and proteins useful as clinical biomarkers. We demonstrated that the use of a peptide signature added predictive value to the Schonberg index that incorporates most important clinical predictors of mortality, and the magnitude of AUC increase (0.042) is in nearly direct proportion to the increase in sensitivity that would be obtained for diagnostic cutoffs at moderately high specificities, as observed in Pencina, D'Agostino & Massaro (2013). Hence, it would represent a not insignificant improvement in diagnostic power of the kind that is often difficult to achieve (i.e., when supplementing a baseline classifier that is already somewhat sensitive) (Baker et al., 2014). Although the magnitude of predictive improvement is modest, it does indicate the potential to develop more robust peptide‐based tools and to use the associations to better understand the biology of mortality. The ability to identify individuals at higher risk well before death, and the underlying molecular disturbances, could provide opportunity to intervene with improved preventive measures. Perhaps most importantly, the improvement in prediction by adding a peptide signature to clinical factors suggests the importance of extraclinical information related to mortality.

The proteins represented by the peptides we found to be associated with mortality reinforce some previous findings that implicate inflammation in the genesis of mortality risk (Reuben et al., 2002; Vidula et al., 2008). For instance, peptides from CRP were strongly associated with the risk of death in our participants, recapitulating many previous reports, and biomarkers of inflammation have consistently been reported for cardiovascular disease and mortality (Weiner et al., 2008). Twenty percent of the mortality‐related proteins in this study were related to regulation of complement activation, an integral element in both adaptive and innate immune systems, that yields the generation of potent inflammatory mediators and cell destruction (Dunkelberger & Song, 2010). Peptides representing proteins in the complement cascade that were increased in men who experienced death within 5 years included peptides of complement factor H, complement C5, and complement C9. Although acute elevations in complement components have been described as predicting poor prognosis among hospitalized patients (Hoesel, Niederbichler & Ward, 2007), our results support the use of these biomarkers to predict mortality in ambulatory men over a relatively long period of observation.

Additional proteins matched to peptides associated with mortality in our analysis are also implicated in inflammatory events. For instance, alpha 1‐antichymotrypsin (AACT) has been associated with Alzheimer's disease, Parkinson's disease, heart failure, and neoplastic conditions (Baker, Belbin, Kalsheker & Morgan, 2007). (Lok et al., 2014; Padmanabhan, Levy, Dickson & Potter, 2006; Zhao et al., 2015; Zhou, Cheng, Tang, Martinka & Kalia, 2016), carboxypeptidase *N* is a plasma zinc metalloprotease mediator of inflammation (Skidgel & Erdos, 2007), and levels of transthyretin have been noted to be depressed in inflammatory states (Ingenbleek & Bernstein, 2015). In fact, we found lower levels of transthyretin peptides in those who died within 5 years and Carriere et al. noted that lower levels of transthyretin were associated with early mortality (Carriere, Dupuy, Lacroux, Cristol & Delcourt, 2008). Hemopexin has been associated with acute inflammatory states such as sepsis (Larsen et al., 2010) and has been considered a potential therapeutic candidate to reduce morbidity and mortality in such conditions (Schaer, Vinchi, Ingoglia, Tolosano & Buehler, 2014). Similarly, vitronectin and kininogen‐1 (Isordia‐Salas, Sainz, Pixley, Martinez‐Murillo & Colman, 2005) are tied to inflammation. The proteins that we identified that are involved in the inflammatory cascade reinforce the importance of the role of inflammation as a presage to mortality and may provide some insight into how they operate in that context over a relatively long time horizon (2–5 years). This constellation of proteins is also consistent with the concept of the senescence‐associated secretory phenotype (Coppe, Desprez, Krtolica & Campisi, 2010), a process considered an important element of aging in which chronic inflammation may derive in part from the secretion of proinflammatory cytokines, chemokines, and proteases from senescent cells. Bioinformatics analyses may provide additional insight. Examining the KEGG pathway for coagulation and complement (Figure 2) suggests that there could be additional biomarkers and contributors to the risk of mortality. In all, our results further solidify chronic inflammation as a harbinger of mortality and suggest specific elements of those pathways that represent elements of the pathway that may be fruitful for further research.

Several other peptides that we found to be associated with mortality are linked to biologically interesting proteins and may be useful biomarkers. We found higher levels of peptides for pregnancy‐associated plasma protein A (PAPP‐A) to be associated with increased mortality. Elevations in PAPP‐A levels are present in acute coronary syndromes (Bayes‐Genis et al., 2001) and have been associated with a number of age‐related disorders, as well as inflammation, prompting a recent suggestion that therapeutic reduction on PAPP‐A may be a strategy to promote healthy aging (Conover & Oxvig, 2017). The biological underpinnings of other peptides for which levels were positively associated with mortality are less clear but may be worth further investigation. A number of the proteins linked to these peptides are involved in cell–cell adhesion and endothelial biology. For instance, VE‐cadherin (CADH5) interacts with several critical signaling pathways (e.g., catenins, fibroblast growth factors, TGF‐β) and plays an important role in endothelial cell biology through control of the cohesion and organization of the intercellular junction (Lagendijk & Hogan, 2015). Leucine‐rich α‐2 glycoprotein 1 (LRG1) is involved in protein–protein interactions, signaling, and cell adhesion (Song & Wang, 2015), and vitronectin contributes to cell adhesion. Vinculin is part of a complex that anchors actin to the cell membrane, participates in cell–cell adhesion, interacts with β‐catenin, and is involved in the control of apoptosis (Saunders et al., 2006). Other proteins associated with mortality in our studies included mast/stem cell growth factor receptor (SCFR or KIT), which is a receptor tyrosine kinase responsive to stem cell factor, is expressed by hematopoietic stem cells and other tissues, and is essential for cell survival by suppressing apoptosis (Lennartsson & Ronnstrand, 2012). Saa4 is a serum amyloid protein of unclear function. In mouse models, it is upregulated by LPS and IL‐6 (Rossmann et al., 2014) and its expression is increased during inflammation, including in muscle during critical illness (Langhans et al., 2014).

Our analysis has important strengths. It takes advantage of a large, prospective observational study that includes excellent follow‐up and ascertainment of mortality. Discovery proteomic measures were performed on almost 2500 men, thus representing the largest such experiment available. Importantly, we included an independent replication phase using targeted proteomics (SRM) to provide added validation of the associations identified in the initial discovery phase. To provide greater assurance that our results were robust, we were able to compare multiple peptide associations at once, demonstrating, for example, that several proteins, such as AACT, A2GL, CO9, CO5, and prothrombin, may be as strongly predictive of mortality as CRP. Demonstrating the association of biomarkers to health outcomes with confidence is challenging, and we included very robust statistical methods to link peptides with mortality risk. Importantly, we replicated findings from the discovery phase with an independent evaluation using targeted proteomic methods (selected reaction monitoring: SRM).

Several limitations should also be mentioned. Our primary proteomic measurements were of peptides and we report those that were robustly associated with mortality. The associations of many were replicated in SRM analyses and they may serve as useful biomarkers. While these peptides can be uniquely linked to specific proteins, not all peptides from any protein were similarly highly associated and thus we cannot comment directly on the levels of intact proteins and their associations with mortality. Moreover, whereas we used rigorous statistical methods to identify the associations of peptides to mortality, in some cases, only one or few peptides from a protein were detected, thus limiting our ability to comment on protein‐level associations. Although the proteomic analysis utilized during discovery is limited in terms of sensitivity, it is also relatively comprehensive and we examined a very large number of participants. We lacked detailed clinical confirmation of the cause of death. As our pathway and protein–protein interaction analyses demonstrate, many of the mortality‐associated peptides we report are from proteins with functions that are biologically linked, and while we can implicate major pathways as being associated with the risk of mortality, it is more difficult to evaluate the relative importance of each peptide/protein. While our results do not definitively point to new clear pathways related to mortality, a number of the associations we detect are unique and suggest avenues for further investigation. Finally, observational studies such as ours are limited in their ability to disentangle the correlative from the causal factors, and from these analyses, we cannot determine the time at which potentially detrimental pathways (e.g., inflammation) become associated with mortality. Future experimental studies may help to elucidate the relationships among proteins and with outcomes relevant to human health.

In summary, we performed large‐scale proteomic analyses on a large number of older men, and describe peptides that are associated with 5‐year mortality. An independent replication study was performed to provide robust validation of these associations. Many of the proteins we identified are matched to proteins involved in inflammatory pathways, suggesting that inflammation is strongly associated with mortality over at least 5 years. These results provide the opportunity to further evaluate these peptides and proteins as biomarkers and highlight the potential importance of the biological pathways they implicate in the origins of death.

## 4 EXPERIMENTAL PROCEDURES

### 4.1 Study participants

MrOS is a prospective observational cohort study of musculoskeletal health in men aged ≥65 years. The design has been described (Blank et al., 2005). Briefly, 5994 community‐dwelling, ambulatory men were recruited from six US communities (Birmingham, AL, Minneapolis, MN, Palo Alto, CA, Pittsburgh, PA, Portland, OR, and San Diego, CA) from March 2000 through April 2002. Eligible participants were at least 65 years old, able to walk without assistance, and had not had bilateral hip replacement surgery. Informed consent was obtained from all participants. The institutional review board at each study site approved the study (review board details provided in Supplementary Materials).

We performed two independent phases of proteomics measures using samples from MrOS participants: an initial discovery phase employed a unique liquid chromatography–ion mobility–mass spectrometry (LC‐IMS‐MS) platform to assess a broad spectrum of serum peptides to identify those associated with mortality, and a replication phase was used to further evaluate discovery phase peptide associations. The replication phase utilized an independent subcohort of MrOS men as well as a targeted proteomic measures (SRM).

#### 4.1.1 Discovery phase

For the discovery phase, 2486 MrOS participants of self‐reported white European ancestry who had sufficient baseline serum available were randomly selected for proteomics analyses. Because non‐white men represented a small proportion of the MrOS cohort (10%), racial comparisons were not possible and we limited our analyses to white participants. There were no significant differences in baseline characteristics of this group compared to the entire MrOS cohort (Table 1). Thirteen samples (about 0.5%) were later excluded owing to technical measurement failures or extreme measurement distributions, leaving 2473 participants in the discovery analysis.

#### 4.1.2 Replication phase

We independently tested the associations with mortality of a set of peptide candidates identified in the discovery phase by assessing the levels of abundance in baseline serum from ~750 men selected from an independent group of 2632 MrOS participants who had not been included in the initial discovery analyses, based on the same selection criteria (white race, adequate serum availability, nonwithdrawal from the study). We randomly selected 533 from the 2632 which contained 54 deaths within 5 years and then enriched the subcohort with samples from the remaining 216 men who died within a 5‐year follow‐up window (control: case ratio approximately 2:1) (Table 1). To check the consistency of peptide abundance levels between discovery and the platform used in the replication phase (SRM), 100 samples chosen at random from the discovery cohort (86 alive + 14 dead) were measured along with the replication cohort. The peptide abundance distributions (standardized within cohorts) were very similar (Figure S2).

### 4.2 Ascertainment of mortality

MrOS participants were contacted regularly during a 5‐year follow‐up period. Information concerning death outcomes was available on >98% of enrolled participants. Deaths were ascertained with the return of a postcard by the participant's family or by a direct contact by study staff and were adjudicated by central examination of death certificates. The cause of death was determined from the death certificate. We considered cancer to be a reliable cause of death from the certificate and included all other causes as noncancer. We focused on deaths within 5‐years, the time point at which sufficient numbers of deaths had occurred in the cohort. However, we also examined 2‐ and 3‐year follow‐up intervals (albeit with fewer observed deaths) to highlight potential relationships with nearer‐term mortality.

### 4.3 Serum proteomic analysis

The sample selection and processing workflow for discovery and replication phases are shown in Figure S3.

#### 4.3.1 Discovery using LC‐IMS‐MS

At all clinical sites, fasting AM blood was obtained and serum prepared using a standardized protocol (Supplemental Material). Serum samples were stored at −80°C until thawed for preparation. For more details of the sample processing and LC‐IMS‐MS analysis, see Nielson et al. (2017). The detected LC‐IMS‐MS features were identified by mapping their mass, elution time, and drift time to an accurate mass and elution time (AMT) tag database using VIPER software tools and linked to proteins. The AMT tag database was constructed using a combination of LC‐IMS‐MS and LC‐MS/MS data generated from serum samples from 151 MrOS participants. Filtering and normalization procedures are described in Nielson et al. (2017). Normalized peptide abundances were used in all subsequent statistical analyses.

In the samples analyzed, we detected 3946 identifiable peptides representing 339 proteins. Peptides present in fewer than 50% of the samples were not considered for further analysis, leaving 2857 peptides (256 proteins) for final analysis in the discovery phase (Figure S3).

#### 4.3.2 Replication using LC‐SRM

SRM analyses were performed using approaches as previously described (Nielson et al., 2016; Shi et al., 2013). A brief method description is provided in Supplemental Material. Of the 56 peptides that were identified as having robust associations with mortality, we chose 21 (representing 16 proteins) for final SRM measurements based on peptide response, transition specificity (co‐eluting interference‐free), detection sensitivity, and LC performance (Figure S3).

### 4.4 Statistical analyses

#### 4.4.1 Discovery phase (LC‐IMS‐MS)

To initially elucidate a set of promising biomarkers for mortality, we employed peptide‐by‐peptide linear regression modeling to estimate age‐adjusted magnitudes of association with (1) all‐cause, (2) cancer, and (3) noncancer death. Our primary objective was to identify proteomic associations with 5‐year all‐cause mortality, but we also estimated associations with mortality within 2 and 3 years. For each peptide, the fold change was estimated by exponentiating the regression coefficient. In the initial discovery analysis, 194 of 2857 (6.8%) peptides demonstrated effect sizes (absolute fold changes) ≥ 1.20 for all‐cause mortality at 5 years, while 348 of 2857 (12.2%) and 261 of 2857 (9.1%) did so at 2 and 3 years, respectively. Ninety‐six (96) peptides were consistent across all three of the time points. As approximately two‐thirds of the deaths were due to reasons other than cancer, peptides selected for noncancer mortality were a subset of those selected for all‐cause mortality. Cancer‐specific mortality analyses yielded very few significant results, likely due to modest numbers of cancer deaths; of these, only two peptides differed from those seen for all‐cause mortality.

These initial effect size estimates were made more robust by means of a cross‐validation procedure implemented using a bootstrap‐approximated jackknife resampling procedure. Briefly, the data were repeatedly split in half randomly, and peptide effects were selected for size in one half but re‐estimated in the other half; the re‐estimated effect sizes were then averaged in a Bayesian framework (Shao, 1989) (see Supplemental statistical methods). We refer to these effect size estimates as *robust*. False discovery rate (FDR) control is inappropriate when using this peptide discovery and cross‐validation pipeline (see Supplemental Materials), but our methods provide adequate control of false discoveries.

#### 4.4.2 Replication phase (targeted SRM)

To determine which peptides would be carried forward from the discovery to the replication phase, we calculated a weighted “importance score” for each peptide based on robust effect sizes and their posterior predictive standard deviations (possible range 0–3; see Supplemental statistical methods). Peptides with importance score greater than 0 were prioritized for replication by targeted SRM; this list was further refined based on suitability of the peptide for SRM assay and optimization of protein coverage. Similar to IMS data, SRM effect sizes were estimated via an age‐adjusted linear regression model on the normalized log‐ratios.

Peptides were declared concordant between discovery and replication phases if they had the same status (positive, negative, or null) in both experiments, and discordant if they had opposite status (either positive/negative or negative/positive) (Supplemental statistical methods).

#### 4.4.3 Peptide signature and mortality

To assess the usefulness of a peptide signature for the prediction of mortality, we examined classification performance using the area under the receiver operating characteristic (ROC) curve and compared the contribution of peptides to a prediction model utilizing the Schonberg mortality index (Schonberg et al., 2009) calculated using baseline MrOS data (Supplemental statistical methods).

#### 4.4.4 Enrichment analyses

To characterize the proteins represented by mortality‐associated peptides for the 5‐year all‐cause endpoint, we conducted Gene Ontology (GO) and KEGG pathway enrichment analyses using the proteins identified as robust in the discovery phase and the workflow described by Schmidt et al. (Schmidt, Forne & Imhof, 2014) (Supplemental statistical methods).

## CONFLICT OF INTERESTS

ESO receives research support from Eli Lilly and Co. and Merck and Co. All other authors have nothing to declare.

## AUTHOR CONTRIBUTIONS

ESO and JL had full access to data and take responsibility for the integrity of the data and the accuracy of the data analysis. JW, CMN, and JL performed data analysis. ESO and JL contributed to study concept and design. ESO, RDS, JJ, and SRC performed data collection/generation. ESO, JW, CMN, and JL drafted the manuscript. All authors revised the manuscript content. EO obtained funding and performed study supervision.
