# Corrigendum

**DOI:** 10.1111/acel.12781

**Published:** 2018-07-19

Orwoll ES, Wiedrick J, Jacobs J, Baker ES, Piehowski P, Petyuk V, Gao Y, Shi T, Smith RD, Bauer DC Cummings SR, Nielson CM, Lapidus J, for the Osteoporotic Fractures in Men Study (MrOS) Research Group. High throughput serum proteomics for the identification of protein biomarkers of mortality in older men. Aging cell 2018 Apr;17(2). https://doi.org/10.1111/acel.12717. Epub 2018 Feb 5.

In the article, “High throughput serum proteomics for the identification of protein biomarkers of mortality in older men,” two sentences (below) in the Discussion erroneously refer to the protein pregnancy‐associated plasma protein A (PAPP‐A) instead of the protein correctly described in Results (alpha2‐macroglobulin (or pregnancy zone associated protein)).

“We found higher levels of peptides for pregnancy‐associated plasma protein A (PAPP‐A) to be associated with increased mortality. Elevations in PAPP‐A levels are present in acute coronary syndromes (Bayes‐Genis, Conover et al. 2001) and have been associated with a number of age related disorders, as well as inflammation, prompting a recent suggestion that therapeutic reduction on PAPP‐A may be a strategy to promote healthy aging (Conover and Oxvig 2017).”

The erroneous text should be deleted from the Discussion.

The authors would like to apologize for the inconvenience caused.

