# Supplementary material for: High‐throughput serum proteomics for the identification of protein biomarkers of mortality in older men
Source: Aging Cell. 2018 Feb 5;17(2):e12717. doi: 10.1111/acel.12717 (PMC5847880; doi:10.1111/acel.12717)
Supplement: Supplementary file 2 [file ACEL-17-e12717-s002.docx]

**Supplemental material**

**Institutional Review Boards, project names and protocol numbers**

University of Alabama at Birmingham Institutional Review Board for Human Use. Osteoporotic Fractures in Men (Mr. OS). F980224007

Human Research Protection Program at the University of Minnesota. Osteoporotic Fractures in Men. 9804M00050

Stanford University Institutional Review Board. Osteoporotic Fractures in Men. 76147

University of Pittsburgh Institutional Review Board. Osteoporotic Fractures in Older Men (MR.OS). 980305

Oregon Health & Science University Institutional Review Board. Osteoporotic Fractures in Men (Mr. OS) Study. 980305

University of California, San Diego Human Research Protections Program. The Osteoporotic Fractures in Men (MROS). 040145

Sutter Health Institutional Review Board. Osteoporotic Fractures in Men. 23.103EXP

University of California, San Francisco Human Research Protection Program. Osteoporotic Fractures in Men. 10-04683

**Sample preparation**

The collection of serum samples at the baseline visit was standardized across the clinical sites. In summary, fasting AM blood was collected by phlebotomy, allowed to clot for 40-45 minutes, centrifuged and frozen at -20C within 30 minutes. Frozen samples were shipped (dry ice) to a central storage facility where they were stored at -80C until analyzed. As recently reported by Hassis et al. this approach should have minimal effects on the validity of proteome analyses (Hassis et al., 2015).

**Methods for selected reaction monitoring (SRM) analyses**

**Peptide selection and preparation**

From the analyses of IMS results, 126 peptides from 74 proteins were selected for SRM measurements based on a priority list generated by a rigorous statistical analysis pipeline (Statistical analyses). Crude heavy peptides for the 126 selected peptides labeled with 13C/15N on C-terminal lysine and arginine were purchased from Thermo Scientific (Rockford, IL) with cysteine modification with carbamidomethylation, and they were mixed together to generate a stock solution with each heavy peptide at a nominal concentration of 5 µM (i.e., 5,000 fmol/µL). To evaluate the peptide quality and select the best responsive transitions for each peptide, 500 fmol/µL of heavy peptide mixtures were subjected to both high-resolution mass spectrometry (MS) (e.g., LTQ-Orbitrap MS) and low-resolution triple quadruple MS analyses with the final three best interference-free transitions for each peptide.

**SRM data collection and analysis**

Patient plasma samples that had been stored at -80°C until use were processed as previously described (Shi et al., 2012; Shi et al., 2013). For each sample the digested peptides from plasma were diluted to 0.25 µg/µL with 0.1% formic acid in water containing crude heavy peptide standards at a nominal concentration of 200 fmol/µL for each peptide. Methods for LC-SRM data collection and analysis are the same as those described previously (Nielson, Jones et al. 2016) except that the retention time scheduled SRM mode was applied. A single scan event was used to monitor the scheduled SRM transitions with scan window of ≥7 min in the scheduled retention time. The average peak area ratios of endogenous light peptides and their heavy isotope-labeled internal standards as obtained from SRM quantification were log-transformed and normalized by incorporating a correction for sample protein concentration, median plate alignment, followed by cubic-spline based smoothing of technical processing variation.

**Normalization of abundance ratios**

Post-experiment normalization of the ratios was achieved using a nonlinear model of attained post-dilution protein concentration. First, the attained well concentration was estimated via tobit regression of the administered dilution amounts on bicinchoninic acid (BCA) assay values previously obtained for the samples. BCA assay values are standardized to a target value of 1.0, so deviations from this value represent surpluses or deficiencies in protein concentration; by subtracting the target value from all BCA observations we obtain a zero-centered target. The coefficient on the centered BCA value is then taken as the empirical dilution ramp. Once the dilution ramp was calculated, the attained concentration was estimated and smoothed using a sigmoidal growth model. Global normalization of the data was performed using Tukey's two-way analysis by medians(Mosteller & Turkey, 1977) on technical control replicates within plates. The global normalization was followed by adjustment for plate characteristics and run date using flexible cubic spline models(Royston & Sauerbrei, 2007) on the median technical control values across plates for each peptide. All analyses were performed on the natural-log transformation of the peptide abundance ratios.

**Supplemental statistical methods**

**Effect size estimation**

The linear regression model for the IMS cohort was:

$$E[y|\mathbf{g}, \mathbf{c}]=\gamma_{1}g_{1}+\gamma_{2}g_{2}+\mathbf{c}'\boldsymbol{\beta}$$

where $g_{1}=1$ indicates alive and $g_{2}=1$ indicates dead. The covariate vector $\mathbf{c}=(age)$, a single scalar. Clinical site was not included in the model because death adjudication was accurate across sites. The fold change in dead participants compared to alive participants of the same age at enrollment is estimated as $\hat{\theta}_{21}={10}^{\hat{\gamma}_{2}-\hat{\gamma}_{1}}$. This model is repeated for all 2857 identified peptides in the reduced target set. In addition to all-cause mortality, we also considered cancer-specific mortality and repeated the analysis pipeline using both cancer death and non-cancer death as the phenotypes of interest. We adopted an inclusive strategy of considering all promising peptides, whether important for all-cause or cancer or both, tagging each peptide with indicators for the generating phenotype.

**Cross-validation**

For cross-validation we used a delete-half jackknife approach (Shao, 1989). The dataset is repeatedly split in half, stratifying by phenotype to maintain the same relative group proportions as in the full cohort. With each iteration, a new half-sample is chosen with replacement. We generated 200 random splits of the data, and for each iteration we used the first half-sample to classify fold changes for candidate peptides as "large" or "small", where "large" is defined as falling within the largest 1/3 of fold changes ranked across all candidates in that iteration: "large" candidates received 1 vote and "small" candidates were penalized by receiving no vote. Effect sizes were estimated in the second half-sample of observations for the "large" candidates only, yielding a set of estimates whose average gives greater expected predictive validity than the naïve full-cohort estimate that may exploit spurious patterns in the data. In other words, we first attempt to determine "large" or "small", and only then calculate a value for "large" — if the result remains large, our initial estimate was right, but if it turns out to be small our initial estimate was wrong and we have uncovered a bias due to heterogeneity in the composition of our cohort. We further refined the small-sample performance of this estimate using a Bayesian shrinkage rule (Gelman et al., 2013) based on an uninformative prior distribution for the log fold change (see Robust effect size estimates).

**Robust effect size estimates**

In Results, we focus on peptides that have a robust effect size of ≥1.2. To achieve robust effect size estimates, the dataset was repeatedly split in half, stratifying to maintain the same proportion of deaths; with each iteration, new half-samples were chosen with replacement. The fold change estimates across the replicates were averaged across 200 iterations using the bias-corrected bootstrap estimate (Efron, 2013), and subjected to a Bayesian shrinkage rule (Gelman et al., 2013) based on an uninformative prior. The appropriate bias-corrected estimate when using the delete-half jackknife (see Cross-validation) is:

$$\hat{\log\theta}^{jack}\equiv2\hat{\log\theta}-\bar{\hat{\log\theta}^{(.)}}$$

where $\bar{\hat{\log\theta}^{(.)}}$ is the ergodic average of the resampled associations (on the log scale) and $\hat{\log\theta}$ is the full-cohort estimate. We refine the small-sample performance of this estimate using the noise variance $\sigma_{0}^{2}$ (which was obtained for each peptide target by estimating the variance of abundances across the pooled technical control replicates added to the experimental runs) in a Bayesian shrinkage rule; this gives us an added check on the volatility of the fold change estimator for noisy experiments such as our that are subject to technology-dependent measurement error that does not improve with increasing sample size. The Bayesian shrinkage estimate $\hat{\log\theta}^{B}$ is then:

$$\hat{\log\theta}^{B}=\frac{\hat{\log\theta}^{jack}\cdot1/{\hat{se}^{2}\left( \bar{\hat{\log\theta}^{\left( . \right)}} \right)}}{1/{\hat{se}^{2}\left( \bar{\hat{\log\theta}^{\left( . \right)}} \right)}+1/{\sigma_{0}^{2}}}$$

This represents our best bias-corrected estimate based on $B$ jackknife resamples. It accounts for confounding in the fold change via regression on important covariates, it accounts for bias in the estimated association (due to heterogeneities in the phenotype disposition) within the cohort via a resampling-based correction, and by shrinking the estimate toward a null prior it accounts for spurious inflation of the effect size due to measurement aberrations that can be influential in small samples. We refer to these effect size estimates as "robust" because the procedure provides a greater confidence in the expected predictive validity of the associations by compensating for unmodeled nonlinearities and biases in the cohort.

**Peptide importance scoring**

After passing through the pipeline, each candidate peptide was tagged with three statistics that could be combined into an overall importance score for the peptide. The first of these was the vote count — the number of times out of the 200 jackknife replicates that the peptide's group effect was considered "large" and estimated. The other two statistics were the bias-corrected fold change estimate and the posterior predictive standard deviation obtained from the Bayesian shrinkage estimator. The log fold change was scaled by the predictive standard deviation to create a predictive z-score; an absolute predictive z-score of 1.65 or larger was taken as evidence that the fold change may be large in a population-average sense. The vote count was converted into a convenient measure of majority support from the voting process. Under this mapping, 0 corresponds to less-than-majority support (fewer than 100 votes, indicating lack of consistency), 1 corresponds to a 2/3 majority, and 2 to a 5/6 majority; any score above 2 indicates nearly universal vote support. To form an overall score, the predictive z-score and majority score were multiplied together. Note that this score includes information about the effect size via the bias-corrected fold change, the significance of the effect via the posterior predictive distribution, and the reliability of the effect via the vote count. An overall score of 1.65 or higher indicates a peptide with at least a 2/3 majority of vote support and a fold change in the highest decile of sizes. We used the maximum overall score across all the tested phenotypes as the selection score for the peptide, and ranked the candidate peptides based on this maximum score. Based on the scoring, the top 329 peptide candidates were selected and proceeded on to validation testing. Approximately 1/3 of these candidates were important only for cancer phenotypes.

**Inappropriateness of false discovery rate correction for filtered selection**

Our peptide discovery and cross-validation pipeline provides an interesting case where false discovery rate (FDR) control is inappropriate. The reason for this is simple: filtration of the input target set leads to bias in the FDR estimator (Efron, 2007). Since we rarefy the set of candidate peptides by insisting the peptides all satisfy a minimum level of attestation in samples and a minimum estimated fold change in the full cohort, and then further reduce the set by deselecting candidates who obtain insufficient votes in the cross-validation procedure, the significance estimates that we obtain by testing hypotheses about the resulting fold changes have a very non-normal distribution, and the null distribution of such scores takes on a skewed nonstandard form. Since we cannot approximate this null distribution well without stronger prior information, FDR–based selection becomes inappropriate in the sense that the FDR estimates would be hopelessly biased.

**Coherence of peptide information**

Supplemental Table 7 presents the discovery-phase support for protein associations based on all the peptide reporters we observed for each protein, SRM replication results where applicable, and the degree to which all the peptides from a protein shared a similar direction of association. The direction coherence score in the table was calculated by summing the absolute values of the z-scores for the peptide associations separately in the positive and negative direction, augmenting each absolute z-score with $1/\sqrt{\#peptides}$ (a continuity correction to reflect the fact that peptide z-scores may fall on either side of zero just due to sampling error even if the true mean is exactly zero), and taking the ratio of the two z-score totals as a precision-weighted "odds" of an individual peptide presenting in the dominant direction. Converting the "odds" to a percentage then yields the coherence score, where values close to 100% reflect a strong tendency for peptides to cluster in one direction. The peptide validation column presents the fraction of peptides successfully replicated in SRM, out of the number selected for replication. To illustrate the dynamics of the selection process, in Supplemental Figure 4 we include an annotated sample forest plot from a random-effects meta-analysis of the peptides mapped to alpha-1-antichymotrypsin (AACT). After screening fold changes of all peptides mapped to this protein, a handful of peptides (6 out of 60) were found with fold changes ≥1.2; of these, most (4 of the 6) were found to be reliable under SRM cross-validation, and the reliable ones were given importance scores based on their effect size, the size of their posterior predictive standard deviations, and the votes acquired during repeated splits of the data. Finally, the peptide with the largest effect size was found to have the largest importance score and also was most amenable to an SRM assay, so this peptide was chosen for SRM validation. Note that the average fold change among AACT peptides was only 1.09, but the vast majority of peptides were in the same direction (coherence score of 87% from Supplemental Table 5), and the largest fold change had a robust estimate of 1.48. Although the magnitude of effect in the selected peptide is much larger than typical for the protein, it is nevertheless in the dominant direction of association and can be viewed as the most *salient* example of the protein's direction of effect (positive, i.e. more abundant in those who died within 5 years).

**Concordance analysis**

Validation of peptide associations with each mortality phenotype was assessed using a concordance statistic measuring the agreement of effect directions between IMS and SRM. For each experiment, we transformed each peptide effect estimate into a Cohen's d-type statistic by scaling the log fold change by a "null" standard deviation:

$$\hat{\eta}\equiv\frac{log(\hat{\theta})}{s_{null}}$$

where $\hat{\theta}$ is the estimated fold change under the technology and $s_{null}$ is the standard deviation of the log abundances in the pooled technical control samples for the corresponding experiment. We set a "null window" $(-e,e)$ for log fold changes on this standardized scale, such that effects in either direction falling within this window would be considered null and not count toward agreement. For IMS we set the half-width of this null window to $e_{IMS}=\left| \log\left( 1.2 \right) \right|$ to reflect the belief that we would be indifferent to seeing an estimated fold change of 1.2 (our minimum value for inclusion on mortality phenotypes) in any peptide with a null standard deviation of 1 in the log abundances. Due to larger technical variance in our SRM data (see above), we set the SRM null window to $e_{SRM}=\left| \log\left( 1.2 \right)/4 \right|$ to compensate for the lower precision. The factor of 4 in the denominator reflects our observation that SRM noise variance tended to be about 4 times larger (relative to the total variance) than IMS noise variance for the same peptide target. Effects were considered "positive" if the standardized effect fell outside the null window in the positive direction ($\hat{\eta}>e$) and "negative" if it fell outside the null window in the negative direction ($\hat{\eta}<-e$); anything within the null window (i.e. $\hat{\eta}\in(-e,e)$) was considered to have "null" status. Peptides were declared concordant between IMS and SRM if they had the same status in both experiments, and discordant if they had opposite status. Ambiguous status determinations such as positive/null or negative/null were given zero weight and disregarded. Concordance was measured using the agreement fraction, conditioning on having unambiguous status:

$$P\left( concordant \right| unambiguous)=\frac{n_{++}+n_{00}+n_{--}}{n_{++}+n_{-+}+n_{00}+n_{\pm-}+n_{--}}$$

This fraction expresses the contribution of concordant peptides to the total number of concordant and discordant peptides. To assess the exact significance of this statistic, taking into account the possibility of achieving large agreement purely by chance perturbations of the marginal distributions, we conducted a permutation test where the SRM status labels are randomly permuted across the peptides and the agreement fraction is calculated for each permutation.

**ROC analysis of peptide signature and mortality**

To assess the usefulness of a peptide signature for the prediction of mortality we examined classification performance using the area under the receiver operating characteristic (ROC) curve and compared the contribution of peptides to a prediction model utilizing the Schonberg mortality index (Schonberg, Davis, McCarthy, & Marcantonio, 2009) calculated using baseline MrOS data. Using logistic regression models we assessed all possible 5-peptide subsets from the 17 concordant peptides that were most predictive for 5-year all-cause mortality in both discovery and validation phases, and selected the best-fitting 10% of models (as measured by AIC) using those subsets. We present the average ROC curve for that 10%. To place the subset models in perspective, a fully nonparametric nearest-neighbor classifier (i.e. taking the mode of the 11 nearest neighbors by Mahalanobis-scaled Canberra distance) that utilized all 17 concordant peptides as predictors could not improve the area under the ROC curve (AUC) compared to the average of the top 10% of 5-peptide subset models, suggesting that such models adequately summarize the combined value of the full set of peptides for predicting 5-year mortality. The percentage of models that each of the 17 peptides appeared in (a simple measure of the importance of the peptide for prediction) is shown in Supplemental Table 4).

**Enrichment analyses**

Enrichment of GO term annotations were evaluated for 30 proteins (the peptide mapped to “Uncharacterized protein KIAA0819 (KO819)” could not be evaluated) using DAVID 6.8 , with knowledge bases updated October 2016. The background for enrichment analyses was the list of 2,934 proteins detectable in the MrOS samples (Supplemental Table 6). Three separate enrichment analyses were performed – for biological process, cellular component, and molecular function GO terms. Analyses of protein-protein interactions (PPI) among the set of proteins associated with bone loss and fracture were done using STRING 10.0 . STRING (Search Tool for the Retrieval of Interacting Genes/Proteins) allowed us to access known and predicted PPI for the set of mortality-associated proteins. STRING derives interactions from among databases of known PPI determined experimentally and from pathway databases, in addition to text mining of Medline abstracts and full-text articles. PPI predictions use co-expression analysis and homology with PPI in other organisms. Both unadjusted p values and FDR values were reported for enrichment and protein-protein interaction analyses.

REFERENCES

Efron, B. (2007). Size, Power and False Discovery Rates. *JSTOR, 35*(4), 26.

Efron, B. (2013). *Large Scale Inference: Empirical Bayes Methods for Estimation, Testing, and Prediction*. New York.

Gelman, A., Carlin, J. B., Stern, H. S., Dunson, D. B., Vehtari, A., & Rubin, D. B. (2013). *Bayesian Data Analysis* (3rd Edition ed.).

Hassis, M. E., Niles, R. K., Braten, M. N., Albertolle, M. E., Ewa Witkowska, H., Hubel, C. A., . . . Williams, K. E. (2015). Evaluating the effects of preanalytical variables on the stability of the human plasma proteome. *Anal Biochem, 478*, 14-22. doi:10.1016/j.ab.2015.03.003

Mosteller, F., & Turkey, J. W. (1977). *Data Analysis and Regression*. Reading, MA.

Royston, P., & Sauerbrei, W. (2007). Multivariable modeling with cubic regression splines: A principled approach. *Stata Journal, 7*(1), 45-70.

Schonberg, M. A., Davis, R. B., McCarthy, E. P., & Marcantonio, E. R. (2009). Index to predict 5-year mortality of community-dwelling adults aged 65 and older using data from the National Health Interview Survey. *J Gen Intern Med, 24*(10), 1115-1122. doi:10.1007/s11606-009-1073-y

Shao, J. (1989). The Efficiency and Consistency of Approximations to the Jackknife Variance Estimators. *Journal of the American Statistical Association, 84*(405), 114-119. doi:10.1080/01621459.1989.10478745

Shi, T., Fillmore, T. L., Sun, X., Zhao, R., Schepmoes, A. A., Hossain, M., . . . Qian, W. J. (2012). Antibody-free, targeted mass-spectrometric approach for quantification of proteins at low picogram per milliliter levels in human plasma/serum. *Proc Natl Acad Sci U S A, 109*(38), 15395-15400. doi:10.1073/pnas.1204366109

Shi, T., Sun, X., Gao, Y., Fillmore, T. L., Schepmoes, A. A., Zhao, R., . . . Qian, W. J. (2013). Targeted quantification of low ng/mL level proteins in human serum without immunoaffinity depletion. *J Proteome Res, 12*(7), 3353-3361. doi:10.1021/pr400178v
